# Increasing Salt Marsh Elevation Using Sediment Augmentation: Critical Insights from Surface Sediments and Sediment Cores

**DOI:** 10.1007/s00267-023-01897-8

**Published:** 2023-11-01

**Authors:** Elizabeth Fard, Lauren N. Brown, Richard F. Ambrose, Christine Whitcraft, Karen M. Thorne, Nathaniel J. Kemnitz, Douglas E. Hammond, Glen M. MacDonald

**Affiliations:** 1 Department of Geography, University of California, 1255 Bunche Hall, Box 951524, Los Angeles, CA 90095 USA; 2 Biological Sciences Department, Bowling Green State University, Bowling Green, OH 43403 USA; 3 Institute of the Environment and Sustainability, La Kretz Hall, University of California, Suite 300, Box 951496, Los Angeles, CA 90095-1496 USA; 4 Biological Sciences Department, California State University, 1250 Bellflower Blvd, MS 9502, Long Beach, CA 90840-9502 USA; 5 U.S. Geological Survey, Western Ecological Research Center, One Shields Ave, Davis, CA 95616 USA; 6 Department of Earth Sciences, University of Southern California, 3651 Trousdale Pkwy., Los Angeles, CA 90089-0740 USA

**Keywords:** Coastal wetlands, Salt marshes, Seal Beach, Sediment augmentation, Grain size, Sea-level rise

## Abstract

Sea-level rise is particularly concerning for tidal wetlands that reside within an area with steep topography or are constrained by human development and alteration of sedimentation. Sediment augmentation to increase wetland elevations has been considered as a potential strategy for such areas to prevent wetland loss over the coming decades. However, there is little information on the best approaches and whether adaptive management actions can mimic natural processes to build sea-level rise resilience. In addition, the lack of information on long-term marsh characteristics, processes, and variability can hamper development of effective augmentation strategies. Here, we assess a case study in a southern California marsh to determine the nature of the pre-existing sediments and variability of the site in relation to sediments applied during an augmentation experiment. Although sediment cores revealed natural variations in the grain size and organic content of sediments deposited at the site over the past 1500 years, the applied sediments were markedly coarser in grain size than prehistoric sediments at the site (100% maximum sand versus 76% maximum sand). The rate of the experimental sediment application (25.1 ± 1.09 cm in ~2 months) was also much more rapid than natural accretion rates measured for the site historically. In contrast, post-augmentation sediment accretion rates on the augmentation site have been markedly slower than pre-augmentation rates or current rates on a nearby control site. The mismatch between the characteristics of the applied sediment and thickness of application and the historic conditions are likely strong contributors to the slow initial recovery of vegetation. Sediment augmentation has been shown to be a useful strategy in some marshes, but this case study illustrates that vegetation recovery may be slow if applied sediments are not similar or at a thickness similar to historic conditions. However, testing adaptation strategies to build wetland elevations is important given the long-term risk of habitat loss with sea-level rise. Lessons learned in the case study could be applied elsewhere.

## Introduction

Climate change presents increasing challenges for the management of coastal wetlands. These wetland ecosystems are considered one of the most heavily threatened natural systems globally (Barbier 2019) and are disappearing at unprecedented rates (Finlayson et al. 2019; Thorne et al. 2018). One of the biggest threats to coastal wetlands in the 21^st^ century is sea-level rise (SLR) (Schuerch et al. 2018). Not only can SLR exacerbate short-term flooding events, but it can also increase the recurrence of inundation periods which may exceed the natural thresholds of coastal ecosystems (Bilskie et al. 2014; Voss et al. 2013). Sea levels are very likely to continue rising over the 21^st^ century, affecting 70 percent of coastlines worldwide (IPCC 2022). Estimates for future SLR rates range anywhere from 29 cm to 1 m by the end of the century, depending on greenhouse gas emission rates (DeConto and Pollard 2016). Global projections anticipate between 20 and 90 percent of coastal wetland loss under low and high sea-level rise scenarios, respectively (Schuerch et al. 2018). This threat is exacerbated by the expansion of coastal development, which leaves many coastal marshes bordered by urban and agricultural usage, preventing marsh migration into adjacent uplands, also known as coastal squeeze (Torio and Chmura 2013). Marsh loss due to SLR, coupled with the lack of upland migration area, will greatly reduce the ability of marshes to maintain their biodiversity, as well as areas of refuge for endemic or endangered wildlife species.

Marsh formation and development results from complex interactions between geologic, hydrologic, and ecological factors that are highly dependent on inorganic sedimentation supply from estuarine sources, especially in areas with little upland inputs (Schile et al. 2014; Byrd and Kelly 2006). In order to develop and maintain optimal elevation levels, salt marshes must have protection from high-energy waves that would erode soil otherwise used for accretion, while also providing source materials (mainly silt, clay, organic matter, infrequently fine sand) from low-wave energy tides (Davidson-Arnott et al. 2002). This sensitivity allows for marshes to respond rapidly to fluctuations in sea level by adjusting their rates of accretion (Adam 1990; French 2006).

Research also shows that coastal marshes can accrete vertically and expand horizontally quite rapidly as a result of storms, including hurricanes (Craft et al. 1993; Schuerch et al. 2012; Thorne et al. 2022). As such, these ecosystems can be reliant on storms to supply sediment, which is useful for quick platform development but may not be sustainable in keeping pace with accelerating SLR (Townend et al. 2011) depending on the marsh location and changes in storm frequency that may deliver sediment for accretion (Thorne et al. 2022). However, Seal Beach has been documented to have limited sediment delivery from storms (Rosencranz et al. 2016; Thorne et al. 2019). In addition, vegetation plays an important role as salt marsh plants reestablish on the sediment surface and aid in marsh accretion through particle capture of fine-grained sediment and aggradation of accumulated vegetation debris, enhancing overall sediment deposition potential (Morris et al. 2002; Leonard and Croft 2006; Perillo et al. 2019). It is important to note that while sedimentation is necessary for a salt marsh’s ability to survive and thrive, an excessive rate of mud deposition can damage the existing vegetation and diminish overall ecological function (Bird 2011; Stagg and Mendelssohn 2010). Few studies have analyzed a thin-layer sediment addition in salt marshes on the Pacific Coast of the United States and even fewer have considered sedimentation rate with historical data, making this research novel and timely.

One approach to address coastal erosion and build marsh elevation relative to sea level has been through sediment addition or augmentation in a variety of ways (Pope 1997; Berkowitz et al. 2017; Ganju 2019). Some approaches place dredged sediments in the nearshore zone with the intent of subsequent tidal or storm redistribution (Schwartz and Musialowski 1980; Fettweis et al. 2011). Sediment addition projects can also focus on augmenting marsh sediment cover by adding dredge materials directly on top of areas of eroding or scouring marsh surfaces to increase marsh elevations relative to sea level. Previous sediment augmentation efforts have been conducted in a number of settings including Essex, UK (Widdows et al. 2006); Venice, Italy (Scarton and Montanari 2015); Narrow River Estuary, RI (Wigand et al. 2017); along the Mississippi River delta region in southern Louisiana (La Peyre et al. 2009; McCall and Greaves 2022); and in various US states including New Jersey (VanZomeren et al. 2018), New England (Perry et al. 2020; Puchkoff and Lawrence 2022), North Carolina (Davis et al. 2022), and California (Thomsen et al. 2022). Such efforts have had mixed results. Widdows et al. (2006) found that, following the placement of fine dredge material (ca. 0.6 m depth) on the upper shore at 2 estuaries situated in Essex, UK, short-term erodibility was high, but long-term temporal changes in sediment erodibility reflected the nature of benthic assemblages established during the recovery period (19 months). La Peyre et al. (2009) found that, following sediment addition at six brackish marsh sites located in the Mississippi River delta region in southern Louisiana, vegetation cover and productivity response were minimal for deteriorating vegetated marshes, with short-term data showing no significant impact of sediment enhancement and long-term trends indicating decreasing productivity over time. While sediment addition or augmentation is not a new approach, the mechanical and fiscal constraints of these large-scale projects have limited the number of examples. As summarized here, the impacts and results of sediment addition have been studied on the East and Gulf Coasts of the United States as well as in other regions of the world. Many of these studies were conducted in microtidal deltaic salt marsh systems where there is some available natural sediment supply. Many fewer, and often more limited, studies are located in urbanized, sediment-limited salt marsh ecosystems in Mediterranean climates like the salt marshes on the Pacific Coast of the United States, making this research critical and timely.

One important challenge that faces ecological conservation and restoration efforts, including sediment augmentation, is the lack of information on the long-term characteristics, processes, and variability in the ecosystem of concern. In many cases, observational data does not extend prior to the period of human alteration. Observational data on past conditions, if available, typically only extends back years to a few decades. Such records cannot capture the naturally variability in the ecosystem (Manzano et al. 2020). The analysis of sediment cores provides one means of furnishing long-term records of pre-disturbance wetland conditions, rates and trajectories of change in physical and biological characteristics, and natural variability in wetland characteristics and processes (Liu et al. 2022; Manzano et al. 2020; Marshall et al. 2020; Gell 2017; Gell et al. 2019).

A sediment augmentation project at Seal Beach National Wildlife Refuge (NWR), southern California, USA provides the first opportunity in this coastal region (Thorne et al. 2018) to test the effect of sediment application to salt marsh surfaces, with the goal of mitigating habitat loss caused by accelerated SLR. The site also provides the opportunity to develop long-term sediment records of marsh characteristics, dynamics, and variability. This region of California contains a number of protected species, therefore acquiring permission to add sediments to existing habitats has been very limited and marsh protection has been a priority. However, threats from SLR submergence have increased the interest of the management community to develop effective adaptive management strategies to build SLR resilience and generate information that is needed to understand the best practices.

In this study, we examine the sediment augmentation and it’s results at Seal Beach NWR in comparison with historic naturally deposited sediments and their depositional dynamics resolved through the use of sediment cores. We examine whether the material used in the augmentation project is similar to sediment found in the current and prehistoric natural environment. We also seek to understand natural accretion and variability over time, and how it compares to the artificial accretion from sediment addition. This type of information is key to inform future sediment augmentation projects. Specifically, we ask (1) What is the grain size and thickness variability of the sediment applied to the newly augmented salt marsh platform? (2) How does applied sediment grain size compare to recent and prehistoric sediment at the augmentation and control site? and (3) How different is the accretion rate of the augmentation layer compared to the natural accretion seen historically in the environment?

## Materials and Methods

### Study Site

**Fig. 1:**
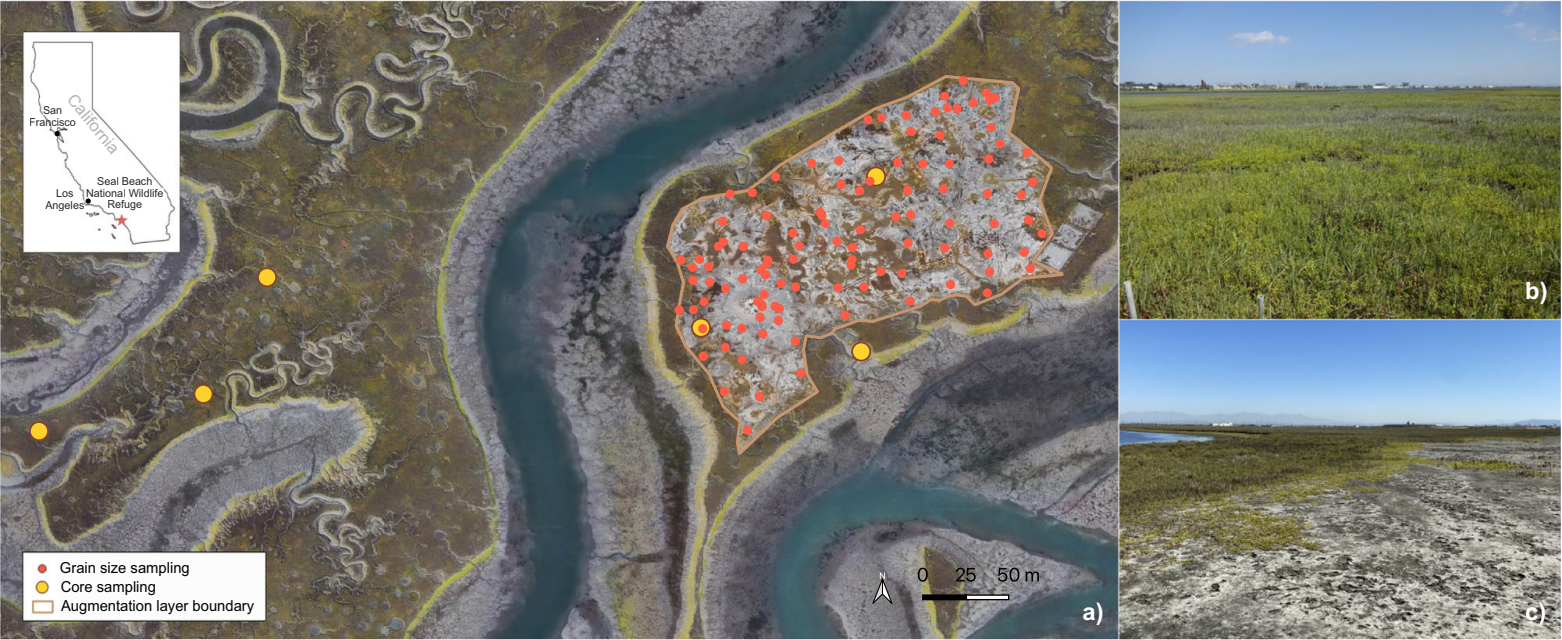
Site map of Seal Beach National Wildlife Refuge, California, USA. With (**a**) surface grain size sample locations and core extraction locations in the augmentation and control site, augmentation site is outlined in red, (**b**) a picture of the marsh plain at the augmentation site in July 2015, and (**c**) a picture of the marsh plain at the augmentation site in March 2022. Background: google satellite in QGIS

This study was conducted at the Seal Beach NWR (Fig. 1), which is managed by the US Department of the Interior, U.S. Fish and Wildlife Service (USFWS). The refuge is located in Orange County, California, USA within the Naval Weapons Station Seal Beach (33˚ 44’ 17.99” N, −118˚ 03’ 60.00” W), and spans 391 hectares, with 304 hectares of tidal marsh, including three intertidal and subtidal restored ponds (McAtee et al. 2020). The refuge consists of approximately 158 hectares of relatively undisturbed salt marshes and is the only remaining undeveloped part of the Anaheim Bay estuary, although surrounded by reclaimed areas of military, municipal, and industrial infrastructure. The climatic and oceanographic settings at Seal Beach NWR are typical of Southern California, with hot/dry summers and mild winters, and semidiurnal tides with a mean micro-tidal range of <2 m (Avnaim-Katav et al. 2017). The marsh harbors state and federally endangered species including the light-footed Ridgway’s rail (*Rallus longirostris levipes*), the California least tern (*Sternula antillarum browni*), and the Belding’s savannah sparrow (*Passerculus sandwichensis beldingi*). Pacific cordgrass (*Spartina foliosa*) and pickleweed (*Salicornia pacifica*) dominate the vegetation landscape, with cordgrass representing 260 ha of the salt marsh platform (Thorne et al. 2019).

Historically, the refuge wetlands received sediment input from episodic storm surges as well as flows from the Santa Ana and San Gabriel Rivers, allowing the refuge to keep pace with SLR in the region (0.98 ± 0.23 mm/yr; NOAA station 9410660) (Grossinger et al. 2011; Rosencranz et al. 2017). Before the twentieth century the salt marsh at Seal Beach became isolated from the Santa Ana River, due to channelization for flood control, therefore making the refuge more vulnerable to accelerated SLR due to a lack of terrestrial sediment inputs (Leeper 2015; Kirwan and Megonigal 2013). Additionally, 4.13 mm yr^−1^ of subsidence has been observed in the region, likely due to oil, gas and water extraction between 1994 to 2012 (Takekawa et al. 2014). The refuge, which is situated along the San Andreas Fault, has also suffered elevation loss due to tectonic subsidence (Leeper 2015). These compounding alterations to the ecosystem and tectonic subsidence, coupled with increased SLR in the region, make Seal Beach NWR especially vulnerable, with one study estimating that the rate of relative SLR is three times higher than that of nearby marshes in the southern California region (Takekawa et al. 2013).

### Sediment Augmentation

#### Construction background

The USFWS designed and implemented the sediment application methodology, along with help from university, state, and federal partners. The Environmental Management Agency, later known as Orange County Public Works (OCPW), managed the dredging, construction of sediment barriers, and sediment application of the project.

The monitoring timeline spanned 6 months prior to the sediment augmentation addition to 5 years post-augmentation. The goal was to uniformly place 10,322 cubic meters of dredged material, thinly spread over 4.05 ha, to achieve a 25 cm (10”) of sediment thickness and to maintain a minimum of 7.6 cm (3”) increase on the marsh platform 2 years after sediment addition (McAtee et al. 2020). The sediment material was sourced from an adjacent subtidal area near the refuge, within Anaheim Bay. Sediment materials from the dredge site were tested for chemical contaminants and grain size compatibility, along with sediment materials from the proposed augmentation site. The proposed dredge materials were deemed to be clean and compatible when compared to the augmentation site materials by Orange County Parks and USFWS (Sloane et al. 2021).

#### Sediment addition

A target thickness of 25 cm was chosen for the sediment addition based on preliminary studies indicating the vegetation recovered rapidly when covered with this thickness of typical wetland sediment. To achieve this thickness, a total of 12,901 cubic meters of dredged material was placed (Thorne et al. 2019). The sediment material was applied in stages, using sediment spray equipment, with the first application occurring between January 22, 2016, and April 4, 2016. A variety of mitigation measures were taken, including relocating rail nesting platforms, maintaining a 50 ft. vegetated buffer zone from the water’s edge, silt barriers around the augmentation site, in-water silt curtains for dredge operations, and maintaining bio-monitors on site (USFWS, pers. comm. Rick Nye). Engineering interventions such as hay bales, straw waddles, sandbags, and geotextile fabrics were placed along the perimeter of the augmentation site to retain the sediment material (Thorne et al. 2019). Dredging challenges arose when obtaining the sediment augmentation material, which appeared to have resulted in sandier grain sizes and lower organic matter compared to the original topsoil at the augmentation site (McAtee et al. 2020).

### Surface Sediment Samples

#### Grain size sampling methods and laboratory techniques

Following augmentation, 113 surface sediment samples were collected and used in this study (Fig. 1). Samples were opportunistically collected immediately after sediment application by USFWS employees (R. Nye, K. Gilligan). Grain size was analyzed for these samples using the Bouyoucos hydrometer method (Bouyoucos 1962), and hydrometer readings and temperatures were recorded immediately (to determine the % sand) and two hours later (to determine the % silt and % clay). We use Bouyoucos’ definitions for sand, silt and clay: sand (2000–50 μm), silt (50–2.0 μm) and clay <2.0 μm.

##### Kriging-based spatial interpolation with grain size

**Fig. 2:**
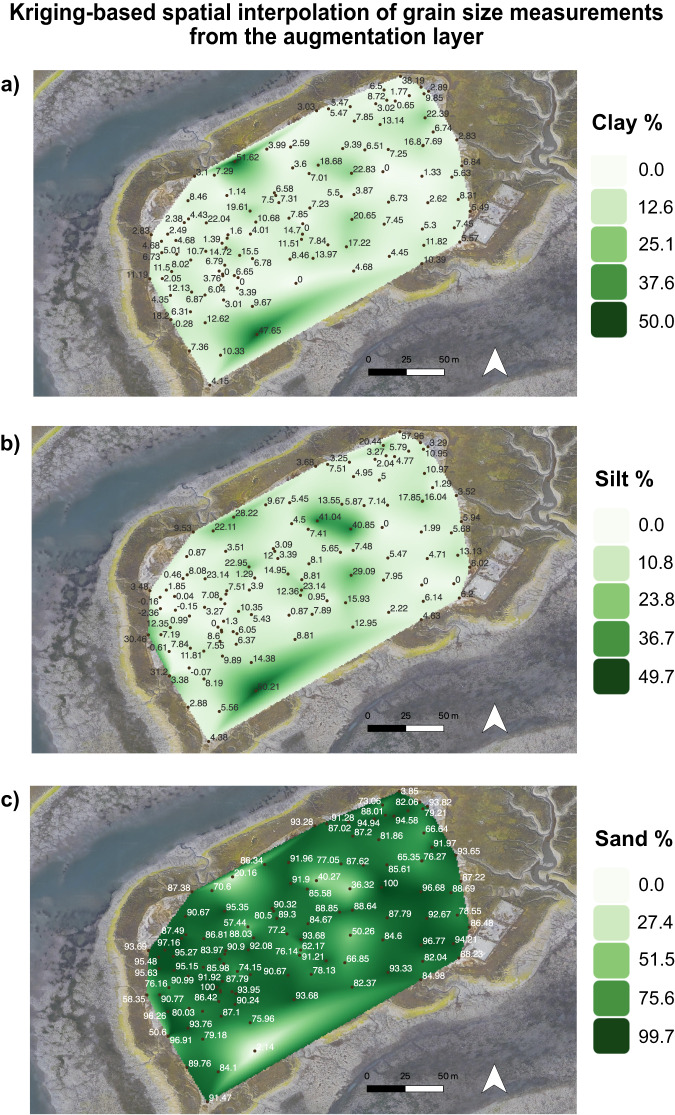
Spatial patterns of grain size in the augmentation layer at Seal Beach NWR, California. Spatial distribution patterns of grain size (**a**) % clay (**b**) % silt and (**c**) % sand. Maps generated by kriging interpolation methods. Background: Google satellite in QGIS

The one hundred and thirteen surface grain size samples were analyzed using the (Sibson) *kriging* interpolation method (Fig. 2). Kriging has been widely used as a geostatistical method in soil science to explore surface variations using spatial correlation methods along a spatially correlated distance (Zhang et al. 2020; Liu et al. 2006; Gotway et al. 1996; Sibson 1980). A total of three maps were created using the Natural Neighbor tool in ArcGIS to visualize the spatial variability of clay, silt and sand values associated with each surface sample taken along the augmentation site following the surface sediment application.

#### Sediment thickness

Measurements of the sediment augmentation thickness were distributed across the entire area with the expectation that the sediment addition would not be uniform, and with the goal of providing representative sampling across the entire area of sediment addition (excluding the buffer area). Although the construction target was even distribution of augmented sediment across the entire project area, spatial heterogeneity was expected. Thus, sediment thickness was sampled at multiple locations across the project area.

Sediment stake stations were established at the Augmentation site. Sediment stakes (also sometimes called erosion pins) have been used widely to determine changes in surface elevations of wetlands (Lee and Partridge 1983; Reed 1989; Kirby et al. 1993; Castillo Segura et al. 2002; Byrd and Kelly 2006; Roegner et al. 2008) and other aquatic habitats (Bradbury et al. 1995). The sediment stakes were 1.9 cm diameter gray PVC pipe with 61 cm buried in the substrate and exactly 55 cm exposed; with a target sediment thickness of 25 cm, this would leave 30 cm of the sediment stake exposed after sediment addition. The sediment stake stations were located on a 20 m grid across the entire sediment augmentation area to ensure even coverage of the site; a wide distribution of sediment stakes provides a good assessment of spatially variable sediment thicknesses. Seventy-one stakes were measured during sampling. Some stakes from the original grid were missing after sediment addition, either because they were inadvertently removed during the sediment addition or because so much sediment was added that the tops of the stakes were buried. The purpose of the sediment stake grid was to provide a more comprehensive spatial assessment of sediment thickness. Because no sediment was added to the control area, a sediment stake grid was not established.

##### Survey timing and field methods

Post-augmentation sampling began in June 2016, two months after the completion of sediment addition. For the first two years, sampling occurred about every six months. The next sampling occurred 12 months later, in 2019, three years after sediment addition. The final sampling occurred in June and July 2021, 62 months after the sediment was added.

Sediment stakes (¾” Schedule 80 PVC pipes) were placed in the sediment with a known height (55 cm) above the substrate. Sediment stakes are commonly used in sediment accretion studies and the protocol is well developed (Roegner et al. 2008). Sediment accretion (accumulation) or erosion was determined by measuring the distance from the substrate surface to the top of the stake. Since all stakes were installed with precisely 55 cm between the ground surface at time of installation and the top of the stake, the thickness of the added sediment was determined as the difference between 55 cm and the measured distance at time of sampling. This length was chosen to ensure that approximately 30 cm (11.8”) would be exposed after the sediment was added to a depth of about 25 cm (10”). Having only 30 cm exposed after sediment augmentation reduced the possibility of predatory birds using the sediment stakes as perching locations.

##### Kriging-based spatial interpolation with sediment thickness

**Fig. 3:**
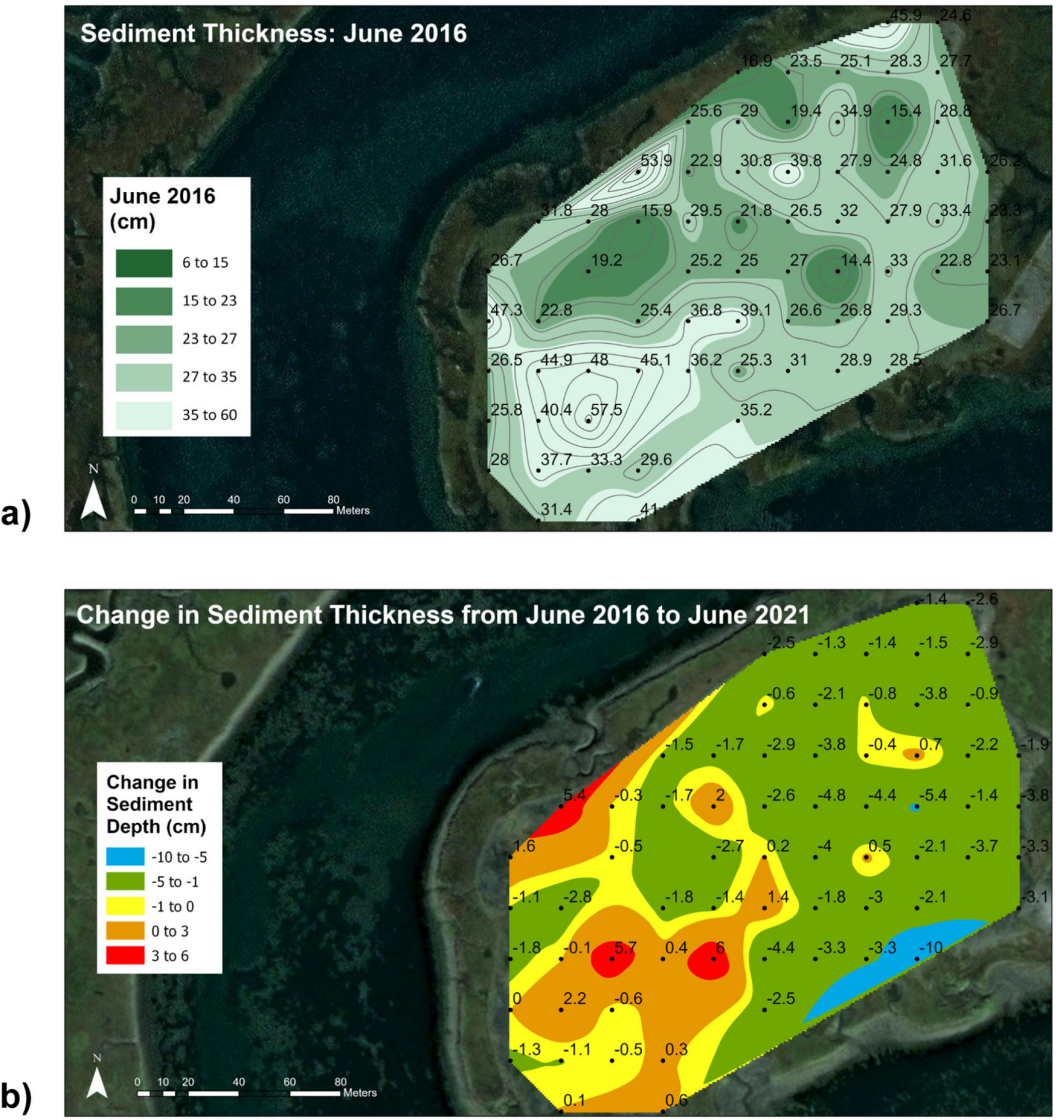
Sediment thickness map. Map of sediment thickness on Augmentation Site for (**a**) 2 months after sediment was added, June 2016 and (**b**) change in sediment thickness over the 5-year period of the study. Data from the sediment stake grid

The fifty-five measured sediment thickness data points were analyzed using the (Sibson) *kriging* interpolation method (Fig. 3). A total of two maps were created using the Natural Neighbor tool and Contour tool in the 3D analyst box in ArcGIS to visualize the five interval classifications for sediment thickness (0–6, 6–15, 15–23, 23–27, 27–35, and 35–60 cm).

### Sediment Cores

#### Sampling site locations and field procedures

Sediment cores were obtained prior to augmentation using a Russian Peat Borer, which takes 1 m lengths of 2.5 cm diameter sediment cores while minimizing compression of sediment samples. Sites were selected in the field with an effort to obtain broad geographic coverage and variation in extant plant coverage (pre-augmentation conditions) on both the control and augmentation sites, while maximizing distance from marsh channels which might have impacted the long-term records due to meandering (Fig. 1). To ensure adequate sampling coverage, material, and replicability, three cores were taken on the control site and three cores were taken from the augmentation site. All cores collected vary from 1 m to 2 m in total. At each core location, a GPS point was taken, and vegetation of the surrounding area was described. All samples were extruded in-field, described, and wrapped in plastic wrap for transport back to the University of California, Los Angeles (UCLA), where they were stored in a cold room at 4 °C.

#### Initial core analysis

Within 10 days of collection, sediment cores underwent initial description and analysis. Cores extruded in the field were unwrapped, photographed, re-measured for any shrinkage or expansion, and visually examined to determine the Wentworth classes for core stratigraphy. Following these preliminary analyses, cores were split in half down to 50 cm depth. One-half of the top 50 cm of each core was sent to California State University Long Beach (CSULB) for analysis of below-ground biomass, while the remaining half was analyzed at UCLA for radiometric activity and carbon content.

#### Chronological control

##### Radiocesium and radiolead preparation

For chronological control over the past century, ^137^Cs and ^210^Pb have been used to determine recent sedimentation (Zhang et al. 2015). These isotopes were used for all six of the cores, and ^14^C dating was used for five of the cores to provide an age-depth model. Based on previous measurements of the ^137^Cs bomb spike depth (1961–63) in Seal Beach sediments, accumulation rates in the area ranged from 2.2–4.6 mm yr^−1^. Consequently, cores were sectioned in 2–4 cm intervals, to a minimum of 20 cm (for low-accreting sites in the high marsh) and a maximum of 60 cm depth (for high-accreting sites in the low marsh). After sectioning, samples were dehydrated in a drying oven at 110 °C for 24 h and then weighed to calculate bulk density (g/cm^3^). Samples were lightly ground, sealed in plastic tubes (1 cm OD, sample heights 2–3 cm), reweighed, and sent to the University of Southern California (USC) for ^137^Cs and ^210^Pb analysis.

##### Excess radiocesium and radiolead

Excess ^210^Pb (^210^Pb_ex_) and ^137^Cs activities in sediments were measured using high-purity intrinsic germanium well-type detectors (ORTEC, 120 cm^3^ active volume). Detector efficiencies were determined by counting standards in a similar geometry. Standards used included IAEA-385 marine sediments, EPA diluted pitchblende (SRM-1), and NIST ^210^Pb liquid solution (SRM 4337). Samples were counted for 2–4 days, to measure the following: ^210^Pb, ^214^Pb, ^214^Bi, and ^137^Cs. Standards were 3.0 cm high, and corrections were made to account for the different sample heights used. The ^226^Ra activity (supported ^210^Pb) was determined from the ^222^Rn daughters (^214^Pb and ^214^Bi). A small (10%) correction was applied to each sample to account for radon leakage, based on measurements of radon loss from similar sediments. Excess ^210^Pb was determined by subtracting the supported ^210^Pb from total ^210^Pb and correcting for decay between collection and analysis.

Two models can be applied to determine sedimentation rates from ^210^Pb_ex_ profiles: the constant rate of supply (CRS) model and the constant initial concentration (CIC) model. Both models assume a time-independent flux of ^210^Pb across the sediment water interface (SWI) and the CIC model also assumes that sedimentation rates are time-independent (Benninger and Krishnaswami 1981; Robbins and Edgington 1975; Robbins 1978; Appleby 2002; Kirchner 2011). For the CRC model, excess ^210^Pb_ex_ inventories were calculated by multiplying excess activity by bulk density and integrating the result downcore. For unmeasured intervals, assumptions were made. Sediments above the top section measured were assumed equal to those in the top interval measured. Linear interpolations were made for deeper gaps. When ^210^Pb_ex_ appeared to be zero for consecutive intervals, the integration was terminated. Error propagation was applied to evaluate uncertainties for missing intervals. Errors for ages determined by the CRC model were calculated by a Monte Carlo approach. Briefly, 1000 random values were generated for each depth interval based on ^210^Pb_ex_ uncertainties for that interval. After the 1000 ^210^Pb_ex_ values were used to determine the interval age, the 1000 ages were averaged, and its standard deviation was calculated. Uncertainties are modest near the top of the core but become quite large as ages reach 2–3 ^210^Pb half-lives. The CIC model gave comparable accumulation rates for each core.

^137^Cs concentrations were often low but gave an indication of the 1961–63 peak from atmospheric weapons testing. A depth range for the age of this horizon was estimated by selecting the observed maximum for the ^137^Cs profile and assuming the actual maximum was midway between this horizon and the subsequent interval.

##### Radiocarbon

For ^14^C dating, organic macrofossil samples for ^14^C were visually identified, extracted from the core, rinsed with DI water, dehydrated in a drying oven at 110 °C for a minimum of 1 h, weighed, wrapped in plastic, and taken to the UC Irvine Keck Radiocarbon Lab for final processing. A total of eight plant macrofossil samples were dated [see Appendix Table 1]. Because any root matter will introduce erroneously young ^14^C ages into older sediments, all plant-matter was identified as above-ground leaves or seeds. Radiocarbon dating was conducted using a 500 kV compact AMS (accelerator mass spectrometer) unit from National Electrostatics Corporation. Plant macrofossil samples and carbonate samples were pretreated following KCCAMS/UCI facilities hydrogen reduction method (Santos et al. 2007). Plant macrofossil organic materials were calibrated using IntCal20 terrestrial calibration curve (Reimer et al. 2020). Age estimates and uncertainties for all ^210^Pb, ^137^Cs, and ^14^C ages were incorporated into a single Bayesian age-depth model using the package rbacon version 2.5.3 with IntCal version 0.1.3 in the R interface (Blaauw and Christen 2013, RStudio Team 2020). All ^14^C ages are reported with 1950 CE as “Present”.

#### Sedimentological analysis

In this study, loss-on-ignition (LOI) was completed for all cores to a depth of 100 cm. Bulk density was also identified, defined as the mass of organic and mineral components, divided by a wet volume of 1 cubic centimeter (Morris et al. 2016). Sediment cores were sliced into 1 cm intervals. From each slice, a 1 cubic centimeter sample was extracted, dehydrated in an oven overnight, burned at 550 °C for 4 h, and at 950 °C for 1 h to measure the water content as a percentage of wet weight, bulk density in grams per cubic centimeter, organic content as a percentage of bulk density, and carbonate content as a percentage of bulk density, following standard protocols from Heiri et al. (2001). Remaining material is interpreted to be the non-carbonate inorganic sediment component.

#### Below ground biomass

Below-ground responses of marshes to environmental factors, such as sea-level rise, have been found to be more broadly applicable than above-ground feedback due to consistency between plants and a lack of dependability on mineral sediment availability (Kirwan and Guntenspergen 2012). Below-ground root biomass, in particular, has been found as an indicator of plant health in marsh environments when compared to above-ground biomass (Turner et al. 2004). The top 50 cm of each sediment core was used to calculate below-ground biomass, with the exception of core SB15_06, which is missing the top 20 cm of sediment. Sediment cores were sieved in 4.75 mm sieves, and small plant roots were rinsed, bathed in fresh water, and dried to remove soil and debris. Dried sieved plant matter (bulk, not separated by type) was then submerged in water in a graduated cylinder to record the volume. Plant roots were then dried, wrapped in pockets of foil, labeled, and placed in a drying oven for 24 h. After drying for at least 24 h at 100–110 °C, the roots were weighed. This measurement is below-ground biomass per unit area (surface area cored).

#### Grain size analysis

For the three sediment cores from the augmentation site and the three sediment cores from the control site, the sampling strategy for grain size aimed to maximize the temporal resolution in the top 1 m (approximately 100–300 YBP). Above 1 m depth, a sample was taken every 2 cm; below 1 m depth, samples were extracted every 5 cm. A total of 255 samples in total were successfully analyzed.

Samples were approximately 0.5 cm^3^ when extracted. They were boiled with 25–30 mL of 30% H_2_O_2_, until reactivity ceased, indicating full removal of organic particles. Samples were then transferred to vials which were transported to California State University Fullerton to the Paleoclimatology and Paleotsunami Laboratory, where they were analyzed using a Malvern Mastersizer 2000 Laser Diffraction Particle Size Analyzer coupled to a Hydro 2000G large-volume sample dispersion unit. Laboratory procedures are further explained in Kirby et al. (2015). Particle sizes were classified as sand, silt, or clay based on the Wentworth scale, which classifies sand as greater than 63 μm, silt between 63 and 3.9 μm, and clay less than 3.9 μm.

Results were plotted using Bayesian age-depth models obtained from R software Bacon (Blaauw 2010) where possible. For those sections of core that were analyzed for grain size, but were below the lowest ^14^C date obtained (or were from a core not ^14^C dated, as in the case of samples from SB15_21), a linear age-depth model was extrapolated by obtaining the average sediment accumulation rate over the Bayesian model (2.1 mm yr^−1^ for SB15_09; 1.9 mm yr^−1^ for SB15_11; 1.7 mm yr^−1^ for SB15_20) or using the ^137^Cs-obtained accretion rate (2.5 mm yr^−1^ for SB15_21). For sediment cores with an age-depth model, the last modeled age was used to start the linear extrapolation.

#### Net sediment accretion rates

Sediment accretion was measured using two methods: feldspar plots and radioisotope analyses of sediment cores. Feldspar plots were created with PVC stakes marking the corner of the plots in the augmentation and control sites before the augmentation sediment layer was added. Feldspar provides a white marker horizon representing the marsh surface before sediment accretion (Cahoon and Turner 1989). To measure sediment accretion rates after sediment addition, additional feldspar plots were established on top of the added sediment by sprinkling (when the plot was exposed to air) 1200–1600 mL of dry Custer Feldspar clay within the perimeter of a 0.5 m by 0.5 m quadrat. The thickness of sediment accumulated on top of the plots was measured by taking a triangular wedge-shaped “core” using a knife and measuring the thickness from the top of the feldspar layer to the top of the sediment; three measurements were taken, one on each side of the triangle, and averaged. If feldspar was visible on the surface of the plot, the thickness was recorded as zero.

Cesium and lead measurements were taken from the sediment cores pre-augmentation. Net marsh sediment accretion rates for the modern period are based on the total depth of marsh sediment accumulated at each core following the 1963 ^137^Cs peak. Longer-term marsh sediment accretion (>60 years) is based upon the total depth of marsh sediment accumulated in each core with the initiation of marsh sedimentation determined by ^210^Pb or ^14^C dating. Depths are divided by time to derive total sediment accretion rates (Fig. 6).

$$Net\,Marsh\,Sediment\,Accretion\,Rate(mm\,yr^{ - 1}) = Depth\,Marsh\,Sediment\left( {mm} \right)/Time\left( {yr} \right)$$

### Permutational Multivariate Analysis of Variance

Permutational Multivariate Analysis of Variance (PERMANOVA) is a non-parametric multivariate statistical test, which does not rely on the assumptions of normality and equal variances. In this study, PERMANOVA was run on the grain size samples to compare (a) the top 10 cm of each core, (b) all the surface grain size samples, and (c) the bottom portion of three cores of which sand represents <20% (SB15_09, SB15_11, SB15_20) compared to the surface grain size samples, to deduce differences between grain size of all the sediments (Anderson 2014; Anderson 2001). The permutational analysis was performed based on the Euclidean Distance similarity matrix. Permutational Analyses of Multivariate Dispersions (PERMDISP) was tested in conjunction with PERMANOVA to identify location vs. dispersion effects, and to look for differences between levels within factors (Anderson and Walsh 2013). All the statistical tests and figures were performed in RStudio Team 2020.

## Results

### Surface Sediment Samples

#### Grain size

A total of 113 surface grain size samples from the post-augmentation surface were used in this study (Fig. 1). Spatial distribution patterns of grain size variability of clay, silt, and sand in the augmentation sediment layer are illustrated in Fig. 2. Light green to dark green on the maps represents concentration levels in percent units. Samples taken of the source material to be added to the Augmentation Site before sediment addition had indicated that the added sediment would be mostly fine-grained, but this preliminary assessment proved to be inaccurate. The grain size of the dredge material contained much less silt and clay (15%) than the pre-sediment application grain size or the control site (57%, 38% respectively) (McAtee et al. 2020). Two months after the sediment was added, 80.1% was sand, 10.7% clay, and 9.2% silt. The sand fraction increased to 89.0% at 62 months, and although this is higher than at two months, the sediment remains dominated by sand (Fig. 2). Unlike the original marsh sediment grain size, the applied sediment and the sediment on the experimental site after sediment application was low in silt and clay content (16%). With 80% of the added sediment being sand (at two months after sediment addition), there wasn’t much opportunity for the sediment to consolidate, shift, or erode into tidal creeks.

The highest percent clay content was located in a small segment at the northern region of the site, as well as throughout a larger segment concentrated along the southern portion of the sediment (Fig. 2a) Similarly for percent silt content, the highest concentrations are found along the southern portion of the sediment, as well as scattered throughout the middle to northern portions of the sediment (Fig. 2b). The largest dissimilarities can be found in comparison with the percent sand content. We also see the contrast between regions with the highest clay and silt percent concentrations, indicated with lighter green shading, and regions with the highest percent sand content, indicated by dark green shading (Fig. 3c). The augmentation sediment layer clearly had higher concentrations of sand through the majority of the site when compared to clay and silt concentrations.

#### Sediment thickness

Two months after sediment was added to the augmentation site, the added sediment had a thickness of 25.1 ± 1.1 cm (Mean ± SE). This is essentially equal to the target thickness of 25 cm. Mean thicknesses varied over time with no clear trend. At 62 months, sediment thickness was 23.9 ± 1.2 cm. The median was lower than the mean for all times, reflecting the influence of a few large values on means. Every sampling period was characterized by a wide variability in thicknesses. Two months after sediment addition, the range was 3.7 to 52.5 cm. The range for successive samples was similar, with a range of 1.7 to 51.9 cm at 62 months.

The spatial variability in sediment thicknesses is illustrated in the thickness contour maps of the sediment stake data (Fig. 3). Two months after sediment addition, there were some distinct areas of thinner and thicker sediment thicknesses. The eastern half of the study site had mostly moderate sediment thicknesses in the 23–35 cm range, although there were a few localized spots with thinner sediment less than 23 cm deep. In contrast, the northwestern quadrant had relatively thin sediment (15–23 cm deep) and the southwestern quadrant had thick sediment (>35 cm deep). This pattern was reinforced over time. The changes in sediment thickness over the five years after the sediment was added show that the eastern side of the study site mostly decreased in thickness, typically losing 1 to 5 cm, while the western side mostly increased, mostly 0 to 3 cm but some portions gained 3 to 6 cm. Most of the changes were modest, either 0–5 cm decrease or 0–3 cm increase, although there were a few isolated pockets of larger changes. Although some areas experienced moderate changes in sediment thickness, the average across the entire site was only a modest decline of about 1 cm from 2016 to 2021.

### Sediment Cores

#### Chronological control

##### ^137^Cs and ^210^Pb_ex_

Average ^137^Cs- and ^210^Pb_ex_-measured accretion for three cores from the augmentation site were 2.9 ± 0.8 mm yr^−1^ and 3.3 ± 0.8 mm yr^−1^ respectively, with average ^137^Cs-measurements showing slightly lower accretion rates compared to ^210^Pb-measurments [Table 1]. Average ^137^Cs- and ^210^Pb-measured accretion for three cores from the control site were 3.9 ± 0.9 mm yr^−1^ and 2.5 ± 0.6 mm yr^−1^ respectively, with average ^137^Cs-measurements showing slightly higher accretion rates compared to ^210^Pb_ex_-measurments. Variation in accretion rates between control and augmentation for all methods was consistently 0.4–1 mm yr^−1^, with the control site average ~0.1 mm yr^−1^ higher than the accretion rate at the augmentation site. The consistency between the sites indicates that these sites are suitable for comparison between vertical accretion as the augmentation study progresses.

**Table 1:** Mean accretion results

		Mean Accretion (mm yr^−1^)		
Site	^137^Cs (n)	^210^Pb (n)	^14^C (n)	All
*Control site*	3.9 ± 0.9 (3)	2.5 ± 0.6 (3)	1.8 ± 0.4 (3)	2.7 ± 1.1 (9)
*Augmentation site*	2.9 ± 0.8 (3)	3.3 ± 0.8 (3)	1.6 ± 0.1 (2)	2.6 ± 0.9 (8)

Control site and Augmentation site mean accretion rates in mm yr^−1^ with standard errors by each method of measuring accretion and across all methods based on ^210^Pb and ^137^Cs, and radiocarbon dates based on ^14^C, for all sampling sites. Accretion rates obtained from USC. Radiocarbon dates were obtained from UC Irvine Keck Radiocarbon lab

##### Radiocarbon

The uncalibrated and calibrated results from ^14^C dating of the six cores appear in Appendix Table 1. Radiocarbon results from the eight samples analyzed for the six cores returned a maximum age of 1502 ± 126 YBP for a 2 m core (SB15_20) taken in the augmentation site, while the youngest date returned was a 1 m core (SB15_06) taken from the control site at 380 ± 78 YBP. By taking an average of long-term accretion rates from ^14^C dates, the estimated average sediment accretion at Seal Beach NWR is 1.7 ± 0.25 mm yr^−1^. Two radiocarbon dates, one for SB15_11 and one for SB15_20, produced anomalously young dates. However, all radiocarbon dates were used to create Bayesian models for all sediment cores which have been ^14^C dated [see Appendix Fig. 1].

#### Sedimentological analysis

**Fig. 4:**
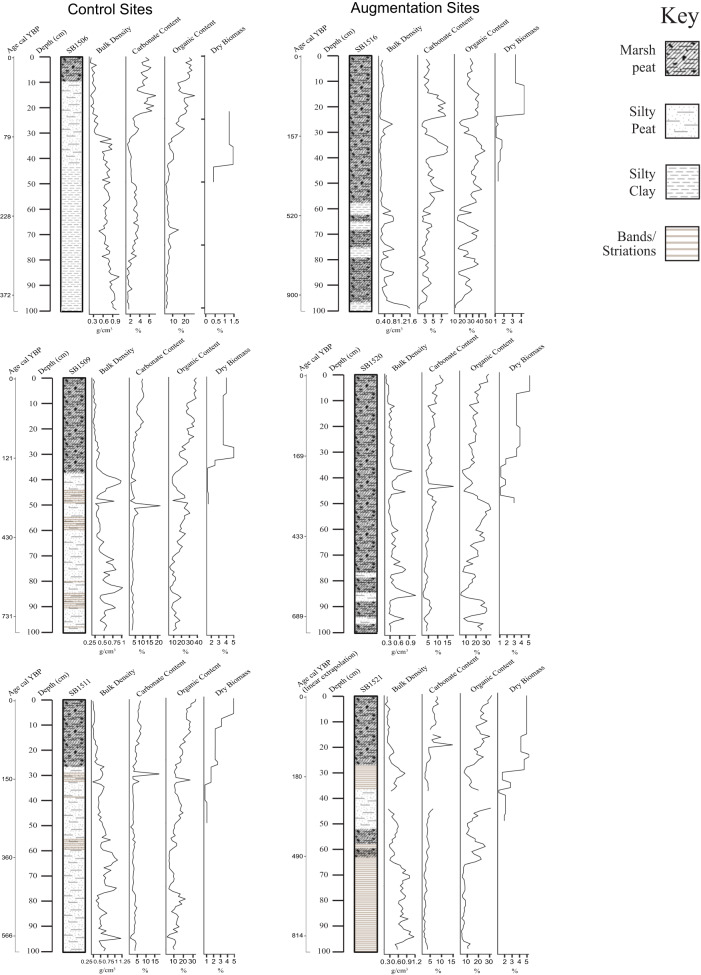
Core stratigraphy. Core stratigraphy, LOI variables (bulk density, carbonate percent, organic percent), and biomass concentrations placed against Depth (cm) and Age. Visual examination was used to determine the Wentworth classes for core stratigraphy

The stratigraphic columns for the top 1 m of each core show that the top 10 cm of each core is indicative of a richly vegetated marsh platform for both sites (Fig. 4). Higher organic marsh peat sections vary, with the augmentation site cores having higher marsh peat segments and the control site cores having more silty peat and silty clay segments throughout the cores.

Bulk density concentrations for all cores (Fig. 4) steadily declined over time. Peak bulk density concentrations are at a maximum of 1.6 g cm^−3^ at around 1000 cal YBP in core SB15_16, with lowest concentrations of 0.1 g cm^−3^ around 20 years ago in core SB15_21. Carbonate content percent has steadily increased in modern times (post-1950s) in all cores apart from SB15_16, which peaked at 49.4% carbonate content around 250 cal YBP and has steadily declined since. Interestingly, the lowest carbonate content is found in the same core at 3.1% around 1000 cal YBP. Similarly, organic content still increased over time for one of the cores from the control site, and the other two cores had high organic content variability at intermediate times (these two cores are also the only cores entirely dominated with marsh peat). For the augmentation cores, more variability is present. Peak organic percent content reaches 21.8% at around 250 cal YBP in core SB15_09, with lowest concentrations of 1.0% around 490 years ago in core SB15_11.

#### Belowground biomass

The vertical profiles of below-ground dry biomass percent for the top 50 cm of each core can be seen in Fig. 4. For all cores, the lowest percent concentrations can be found towards the bottom of the cores. For SB15_06, below-ground biomass percent peaks at 1.5% between 97 and 130 cal YBP, and lowest concentrations of 0.4% are between 135 and 163 cal YBP. For SB15_09, below-ground biomass percent peaks at 5.0% between around 93 and 131 cal YBP, and lowest concentrations of 1.5% are between 170 and 304 cal YBP. For SB15_11, below-ground biomass percent peaks at 4.9% at the top of the core in the very recent past (between 2015 and 1976), and lowest concentrations of 0.8% are between 175 and 215 cal YBP. For SB15_16, below-ground biomass percent peaks at 4.3% at the top of the core between 1970 and 78 cal YBP, and lowest concentrations of 1.1% are between 135–180 cal YBP. For SB15_20, below-ground biomass percent peaks at 5.2% at the top of the core in the very recent past (between 2015 and 1989), and lowest concentrations of 1.1% are between 276 and 301 cal YBP. For SB15_21, below-ground biomass percent peaks at 5.3% at the top of the core between around 111 and 121 linearly extrapolated cal YBP (24−25 cm depth), and lowest concentrations of 1.2% are between 251 and 261 linearly extrapolated cal YBP (38–39 cm depth).

#### Grain size analysis

**Fig. 5:**
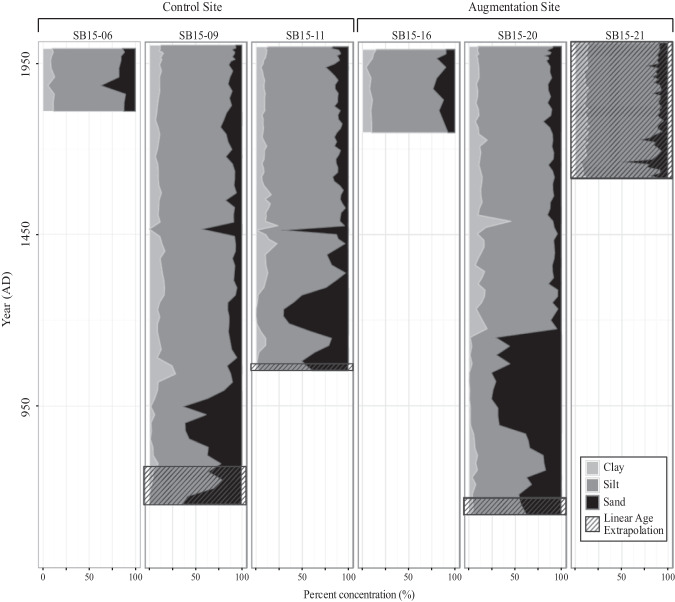
Core grain size. Grain size analysis by time for the control and augmentation sediment cores

Results show that pre-augmentation grain size as represented by the top 5 cm of the cores averages 11% clay, 77% silt, and 10% sand (Fig. 5). When comparing the top 10 cm of the six cores, we see that historical grain size values are fairly consistent across cores, between the augmentation and control sites [Table 2]. Similarly, there is consistency between our three longer cores around 1450 AD and older (Fig. 5). The maximum sand percentage increases down-core and the highest measured in any sample analyzed was 76%, in core SB15_20. Of the six cores analyzed, three cores (SB15_09 (135–200 cm), SB15_11 (125–180 cm), SB15_20 (115–220)) show periods of high sand concentration (>20%) below 1450 AD where habitat may or may not have been salt marsh as it is today.

**Table 2:** PERMANOVA results

ID	df	SS	MS	Pseudo-F	P(perm)	Perms
Top 10 cm of cores (6)	5	176.85	35.371	2.1841	0.073	999
Res	18	291.5	16.194			
Total	23	468.35				
Top 10 cm of cores (6) to augmentation	1	196482	196482	503.88	0.001	999
Res	136	53031	390			
Total	137	249514				
Core segments with sand > 20% (3) to augmentation	1	94662	94662	188.26	0.001	999
Res	160	80450	503			
Total	161	175112				

PERMANOVA results table for grain size comparisons include Top 10 cm of cores (6 cores used), Top 10 cm of cores (6 cores used) to the augmentation layer (113 surface samples), and Core segments with sand >20% (3 cores used) to the augmentation layer (113 surface samples)

*df* degrees of freedom, *SS* sum of squares, *MS* mean sum of squares, *Pseudo-F* *F* value by permutation, *P(perm)* *p*-values based on more than 999 permutations (the lowest possible *p*-value is 0.0001), *Perms* number of permutations

The above results compare to post-augmentation grain size measurements taken from February - June 2016, which averaged 9% clay, 10% silt, and 83% sand. Although clay concentrations remained relatively similar in the pre-augmentation and post-augmentation sediment materials, the sand concentration found at the site post-augmentation greatly exceeds sand concentrations at the top of the cores in both the control and augmentation sites (pre-augmentation), as well as any sand concentration obtained in analysis of all cores covering a history of 1500 years of accretion.

By plotting the grain size results by age (Fig. 5), we can estimate that the lenses seen in cores SB15_09, SB15_11, and SB15_20 are an event previously identified as an abrupt subsidence event due to a tectonic event caused by the nearby Newport-Inglewood/Rose Canyon fault system (Leeper et al. 2017). Leeper et al. identify this event as having occurred from approximately 1320 AD to 1590 AD. This matches the increase in larger particle sediment seen at approximately 1450 AD in the three cores identified above. It is also possible that the lens seen in SB15_21 corresponds to this event, but because it lacks a Bayesian age-depth model the linear age-depth model underestimates the age of this event. This is very probable, as accumulation rates tend to decrease with depth, so using ^137^Cs-based accumulation rates tends to underestimate age below the cesium peak. Further ^14^C dates around this area would resolve this question.

#### Sediment accretion

During the first year after sediment addition, nearly all of the plots on the augmentation site still showed feldspar on the surface, indicating negligible sediment accumulation. By one year after the sediment was added, an average of 0.5 mm of sediment had accumulated; this average was driven by a few plots with 2–3 mm of sediment accumulation, but most plots still had feldspar showing on the surface. Sediment slowly continued to accumulate until there was an average of 5.9 mm of sediment on top of the feldspar layer 62 months after sediment addition, an average accumulation of 1.2 mm/yr. There was a very wide range in accumulation, with a few plots showing none while one plot showed 23 mm. At the control site, the average sediment depth was 14.3 mm one year after sediment was added to the augmentation site. After this rapid increase in the first year, accumulation decreased, with sediment accumulation reaching 18.9 mm at 62 months, an average of about 3.9 mm/year at the control site.

The mean accretion rates with standard errors by each radioisotope method of measuring accretion and across all methods from the control site and the augmentation site are shown in Table 1. For ^137^Cs, the mean accretion rate is 3.9 ± 0.9 mm yr^−1^ at the control site, and 2.9 ± 0.8 mm yr^−1^ at the augmentation site. For ^210^Pb_ex_, the mean accretion rate is 2.5 ± 0.6 mm yr^−1^ at the control site, and 3.3 ± 0.8 mm yr^−1^ at the augmentation site. For radiocarbon (^14^C), the mean accretion rate is 1.8 ± 0.4 mm yr^−1^ at the control site, and 1.6 ± 0.1 mm yr^−1^ at the augmentation site. For total mean accretion rates (as determined from ^137^Cs, ^210^Pb_ex_, and ^14^C dating), the mean accretion rate is 2.7 ± 1.1 mm yr^−1^ at the control site, and 2.6 ± 0.9 mm yr^−1^ at the augmentation site, with consistency between control and augmentation sites and radiometric methods.

**Fig. 6:**
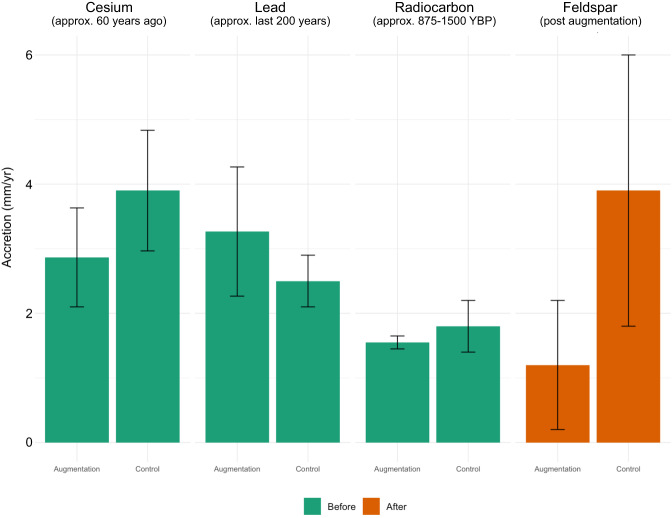
Accretion rates by method. Comparison of vertical sediment accretion rates by method of collection. “Before” signifies sediment accumulation before the application of the augmentation layer; “after” refers to sediment accumulation after the augmentation layer was applied

Comparison of vertical sediment accretion rates by method of collection and with reference to before or after application of the augmentation sediment layer can be seen in Fig. 6. While there are smaller dissimilarities between accretion rates at both the control and the augmentation site before the sediment layer was added, the largest contrast can be seen in feldspar mean accretion measurements that were taken after the augmentation sediment layer was added to the site. Sediment accretion in the control site after sediment was added was similar to the ^137^Cs accretion rates, whereas accretion in the augmentation site was much lower than the ^137^Cs accretion rates, although there was a lot of variability among samples in the post-augmentation data.

### Permutational Multivariate Analysis of Variance

PERMANOVA tests can be seen in Table 2. One test compares grain size samples for the top 10 cm of each of the six cores. The second test compares grain size samples for the top 10 cm of each of the six cores to all the surface grain size samples from the augmentation site. The last test compares the bottom portion of three cores in which sand represents >20% (SB15_09, SB15_11, SB15_20) to the surface grain size samples from the augmentation site. A multivariate dispersion model was performed to test whether the groups had homogenous dispersion. For the first model (the top 10 cm of each core), the multivariate dispersion model showed that groups had homogenous dispersion, therefore suggesting that the result is indeed driven by differences in the centroids. The null hypothesis of homogenous dispersion was not rejected for models 2 and 3. However, this could be due to the unbalanced nature of our sample groups (Anderson 2001).

PERMANOVA tests comparing the top 10 cm of each core reveal a lack of significant differences between the cores (*p* = 0.073, R^2^ = 0.38). However, PERMANOVA tests comparing the top 10 cm of the cores to the newly added augmentation sediment yielded significant differences (*p* < 0.001, R^2^ = 79% of the variation in distances explained by the groups). Similarly, PERMANOVA tests comparing the core segments with sand >20% (SB) to the augmentation sediment layer yielded significant differences (*p* < 0.001, R^2^ = 54% of the variation in distances explained by the groups).

**Fig. 7:**
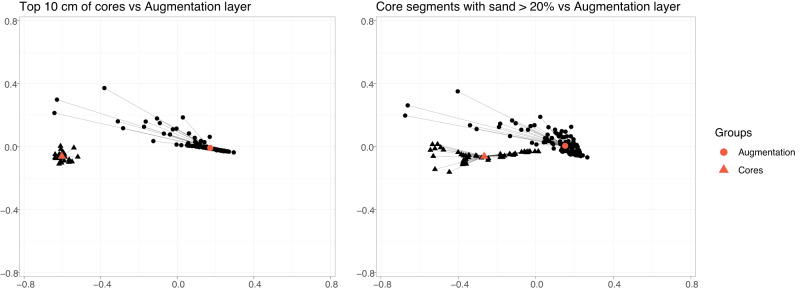
PERMANOVA distance matrix. PERMANOVA centroid and distance matrix results visualized for the top 10 cm of cores compared to the augmentation sediment layer, and core segments with sand > 20% compared to the augmentation sediment layer

The Pseudo-F value for the top 10 cm of cores compared to the augmentation layer is higher than the core segments with sand >20% compared to the augmentation layer (503.9 and 188.3, respectively). This larger pseudo-F value suggests that there are greater distances in our comparison between the top 10 cm of the cores and the augmentation layer, and lower distances in our comparison between the core segments with sand >20% and the augmentation layer sediment material. These differences are visualized in Fig. 7, which shows the centroids of the augmentation layer compared to the top 10 cm of cores as well as the core segments with sand >20%. An important conclusion that can be drawn from the statistical analysis is that the augmentation sediment is significantly coarser in terms of sand content than even the most coarse natural sediments found in the lower portions of the cores.

## Discussion

### Historic Conditions versus Augmentation

Applied sediments were markedly different from prehistoric sediments at the site in composition of sand. While the disparity between augmentation grain size and natural grain size is concerning, this record of rapid environmental change demonstrates a potential capacity for recovery. By plotting the grain size results by age, we can estimate that the lenses seen in cores SB15-09, 11, and 20 are an event previously identified as an abrupt subsidence event likely due to a tectonic event caused by the nearby Newport-Inglewood/Rose Canyon fault system (Leeper et al. 2017). Leeper et al. identify this event as having occurred from approximately 1320 AD to 1590 AD. This matches the increase in larger particle sediment seen at approximately 1450 AD in the three cores identified above. Similarly, changes between a sand-dominated grain size environment and a silt-clay dominated grain size environment have occurred in the past on estimated timescales of 10–30 years, as well as historically in the early phases of marsh formation (Fig. 5).

This difference demonstrates that sand concentration post-augmentation greatly exceeds sand concentrations at the top of the cores in both the control and pre-augmentation sites, as well as exceeds any sand concentration obtained in the analysis of all cores covering a history of 1500 years of accretion. Additionally, we see differences that are statistically significant when comparing the augmentation layer to the top 10 cm of the cores, as well as the core segments with >20% sand concentrations. These results confirm that the sediment material in the augmentation layer is distinct from the grain size material of the natural environment found at any point in time at the site. Given this range of measured historic conditions, there may be a potential for this marsh to revegetate and trap more fine-grain sediments which could return it to a similar state prior to augmentation. Long-term monitoring will be key to following this possible recovery.

### Accretion Rates

We measured accretion rates using three radiometric approaches. Because Seal Beach NWR is cut off from upland freshwater inputs by human development all sediments for accretion are marine or aeolian inputs, or intra-marsh redistribution of mineral material, or organic matter contributions. Rosencranz et al. (2016) reported very low sediment flux import for Seal Beach NWR. However, historically, measured accretion rates at Seal Beach NWR were fairly typical of the region (Brown et al. 2022; in-prep, Thorne et al. 2018). These historic accretion rates for ^137^Cs and ^210^Pb are similar or on the low end for North American salt marshes, which can see vertical accretion anywhere from 1 mm yr^−1^ to 10 s of mm a year in high-accreting zones (Kirwan et al. 2016).

Similarly, by taking an average of long-term accretion rates from ^14^C dates, the estimated average sediment accretion at the augmentation site was 1.6 ± 0.1 mm yr^−1^, and 1.8 ± 0.4 mm yr^−1^ at the control site [Table 1]. These values are typical for accretion rate measurements obtained from ^14^C-dating in North American salt marshes (Holmquist et al. 2021), especially those on the Pacific Coast (Thorne et al. 2019). ^14^C accretion rates are, however, lower when compared with ^137^Cs, ^210^Pb accretion rates, or modern monitoring methods such as feldspar marker horizons. This is presumably due to the time span this analysis covers. Natural processes such as sediment compaction, local subsidence, and organic decay make dating methods over longer timespans, like ^14^C rates of accretion, an underestimate of current rates, and therefore unsuitable for comparison use in modern ecosystem monitoring (Mudd, Howell and Morris 2009).

Following sediment addition, the difference in accretion rates between the augmentation site and the control site may be explained by tidal flooding and vegetation cover. Tidal flooding delivers suspended sediment particles to bolster marsh accretion which can be related to time flooded and depth (Temmerman et al. 2005). The elevation of the augmentation site after construction was about 45 cm (NAVD 88) higher than the control site (McAtee et al. 2020), reducing time flooded by tides. Tidal inundation can also disperse seeds and rhizomes to areas for plant establishment in marshes (Rand 2000).

### Adaptive Management

Seal Beach NWR habitats have been documented as being at risk of submergence from accelerating SLR (Takekawa et al. 2013; Thorne et al. 2016), and sediment augmentation was identified as a possible strategy to reduce potential habitat loss over the long term (Thorne et al. 2019; Rosencranz et al. 2019). Near-term (1 year after application) negative impacts on the vegetation and invertebrate community were observed following the project (McAtee et al. 2020). The application site prior to sediment addition had a diverse assemblage of plants with generally high cover, but low stature, therefore, it was not optimal habitat for endangered nesting birds such as the light-footed Ridgway’s rail (*Rallus obsoletus levipes*) and Belding’s savannah sparrows (*Passerculus sandwichensis beldingi* (Fig. 1b). Both of these species have been identified as being impacted by habitat loss with SLR (Rosencranz et al. 2018; Rosencranz et al. 2019), and managers were keen on conducting case studies to prevent extinction. Vegetation recovery has been slower than predicted by preliminary experiments (Rishi 2014; Sloane et al. 2021). This manuscript hypothesizes that slow vegetation recovery could be due to the composition of the dredged material compared to original marsh soils. Our results show that, due to the differences in sediment grain size of the parent material compared to the newly added outsourced material, the sand fraction is higher and the organic content of the sediment is much lower at the augmentation site. In a depositional environment like the salt marsh at Seal Beach, small particles such as silt and clay tend to make up the dominant portion of mineral material. Ideally, grain size added during thin layer sediment application to increase elevation should be similar to grain sizes seen in the past to mimic natural salt marsh conditions and promote plant growth. In addition, increasing the marsh plain (in this case by 25 cm) will result in changes to hydrology and sediment dynamics. One consideration is that slow recovery of vegetation could be a potential tradeoff when building vertical resilience. However, in the long-term, this higher marsh plain may reduce the vulnerability to drowning by SLR.

Seal Beach NWR required urgent intervention due to the level of drowning of the marsh platform in recent years. Time constraints combined with geographic limitations on access to appropriate sediment sources played a role in slow recovery rates. The lack of access to sediment sources such as fluvial networks, storm events, or more likely organic matter accretion coupled with the newly developed supratidal elevation regions also reduced the tidal inundation period across the augmentation site. Additionally, reduced tidal inundation can influence salinity levels. High salinity concentrations impede plant communities from establishing, reducing the ability of crustaceans, mollusks, and other biota to move in and thrive, preventing the microtopographic salt marsh landscape from developing and aiding in marsh resilience (Whitcraft and Levin 2007; Sievers et al. 2019). These conditions make it difficult to predict marsh recovery time horizons and highlight the importance of having a historical lens of sediment characteristics to try and mimic natural processes for successful recovery of salt marsh habitat. Furthermore, post-treatment monitoring is crucial for providing insight into the potential effects of augmentation on long-term marsh recovery.

## Conclusion

The observed changes at SBNWR reflect a combination of interrelated factors and processes: the thickness of the sediment applied, the resulting supratidal elevation, harsh abiotic parameters, dispersal inhibition, and the characteristics of the dredge material (McAtee et al. 2020; Sloane et al. 2021). The artificial application of thin-layer sediment at Seal Beach NWR marsh is one of the first attempts to maintain marsh habitat with sediment enrichment along the Pacific coast of the USA. This study at a Pacific Coast marsh provided a unique opportunity to understand the impacts of sediment augmentation within the long-term dynamics of the marsh as revealed by sediment cores and shorter-term response to the augmentation as revealed by post-treatment surveys. The results of the study indicate that although the marsh experienced appreciable variability, in pre-historic sedimentary and biological characteristics, the nature of the coarse-grained augmentation sediment and the thickness of application had no counterpart in the natural variability of the marsh. This mismatch likely contributes to the initial relatively slow recovery of a vegetated state. On this basis, we would recommend that analysis of sediment cores should be an important part of sediment augmentation and marsh restoration planning.

### Supplementary Information

Appendix Figure1

Appendix Figure2

Appendix_Table1

Appendix

## Data Availability

Data is available upon request. Please contact corresponding author.
