# Supplementary figures and images for: Increasing Salt Marsh Elevation Using Sediment Augmentation: Critical Insights from Surface Sediments and Sediment Cores
Source: Environ Manage. 2023 Nov 1;73(3):614–33. doi: 10.1007/s00267-023-01897-8 (PMC10884093; doi:10.1007/s00267-023-01897-8)

# Bacon Age-Depth Models for Sediment Cores

a) SB15-06

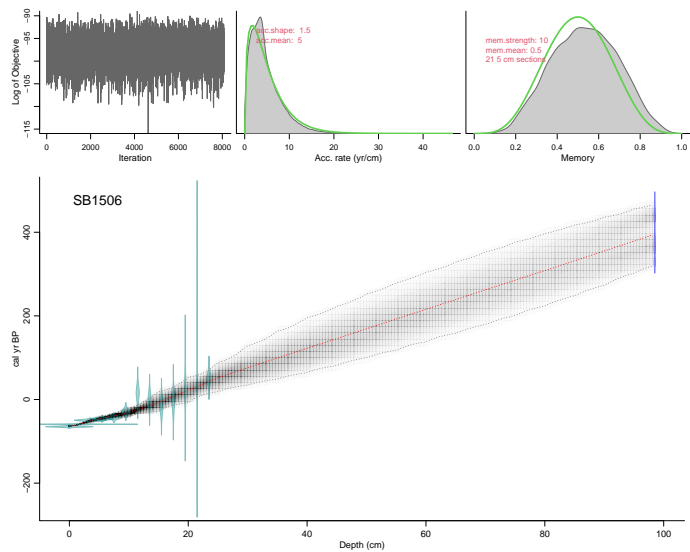

b) SB15-09

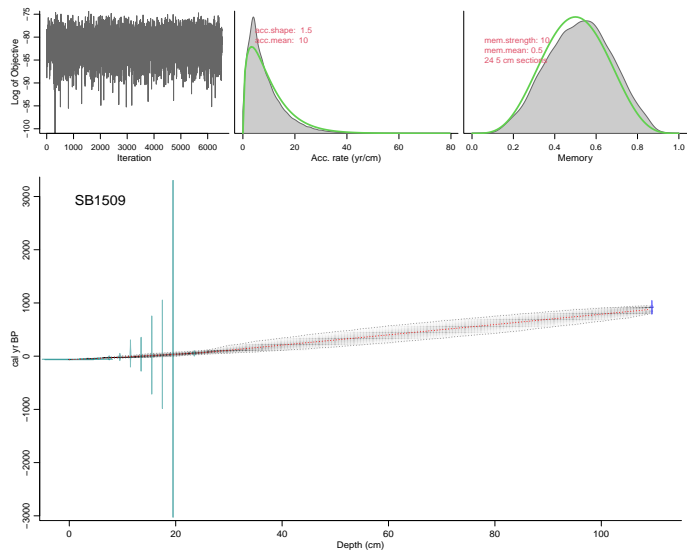

c) SB15-11

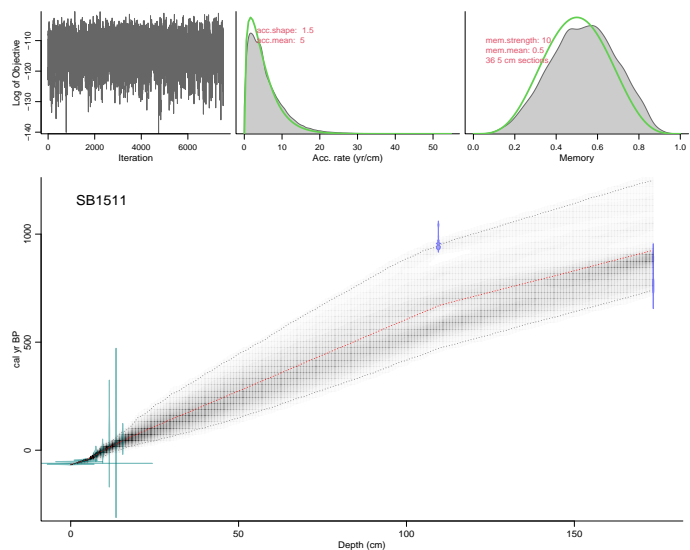

d) SB15-16

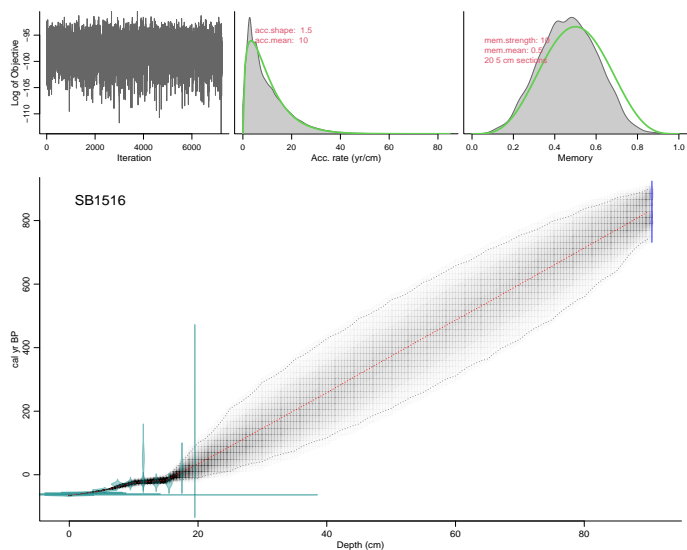

e) SB15-20

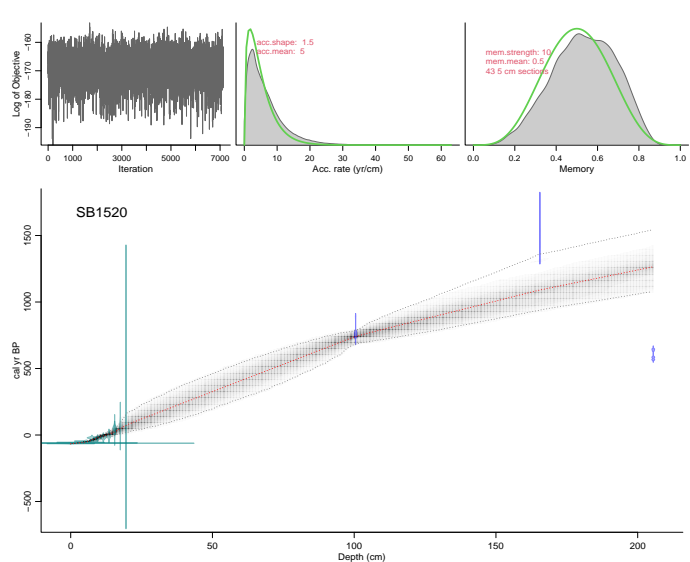

f) SB15-21

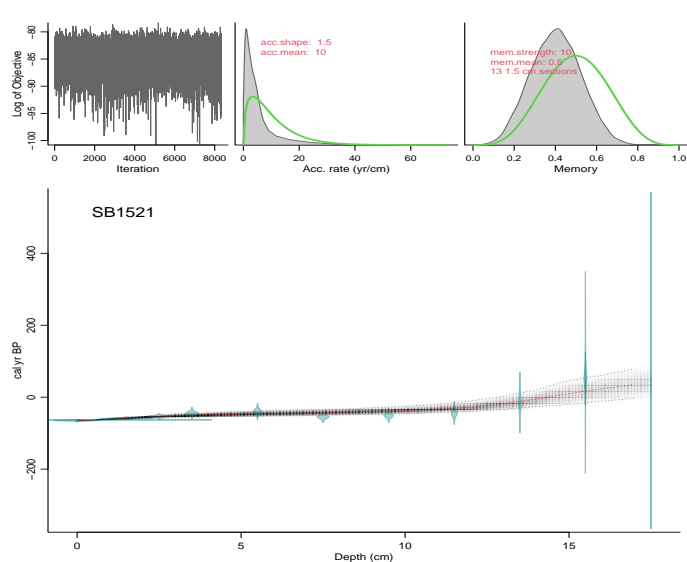

Supplement: Supplementary file 1 — Appendix Figure1 [file 267_2023_1897_MOESM1_ESM.pdf]

# Lead and Cesium Curves for Sediment Cores

a) SB15-06

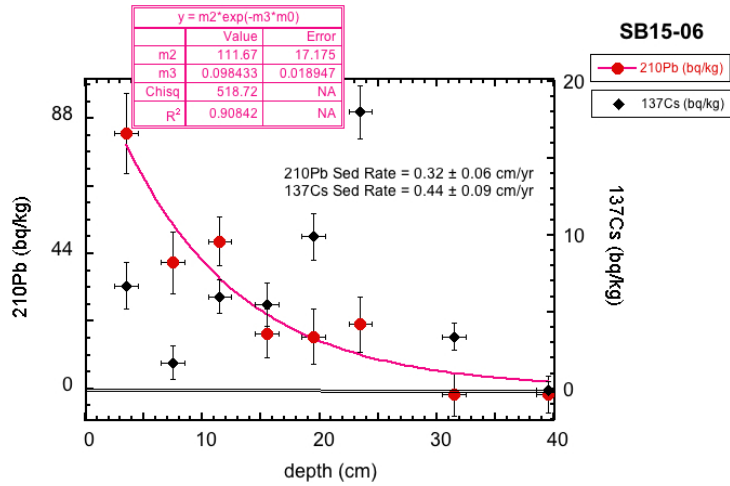

b) SB15-09

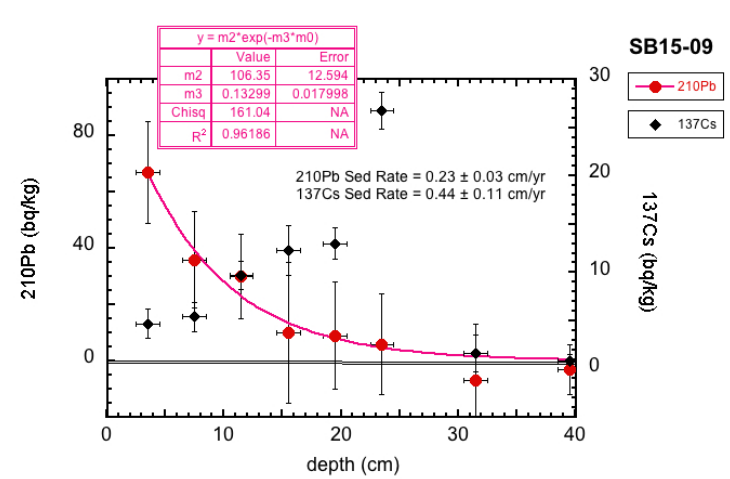

c) SB15-11

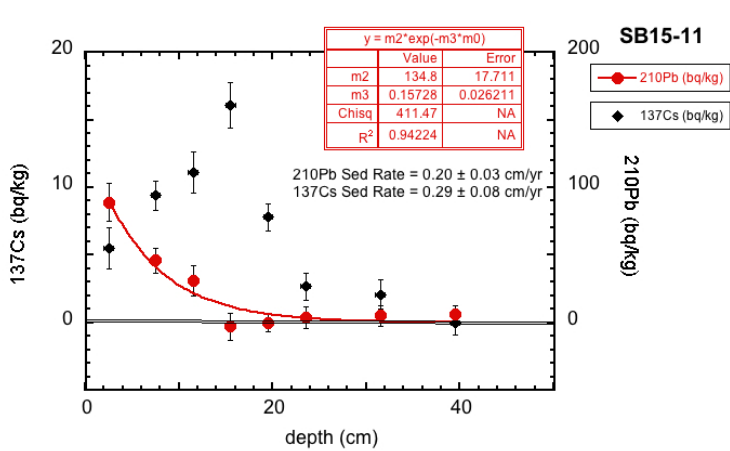

d) SB15-16

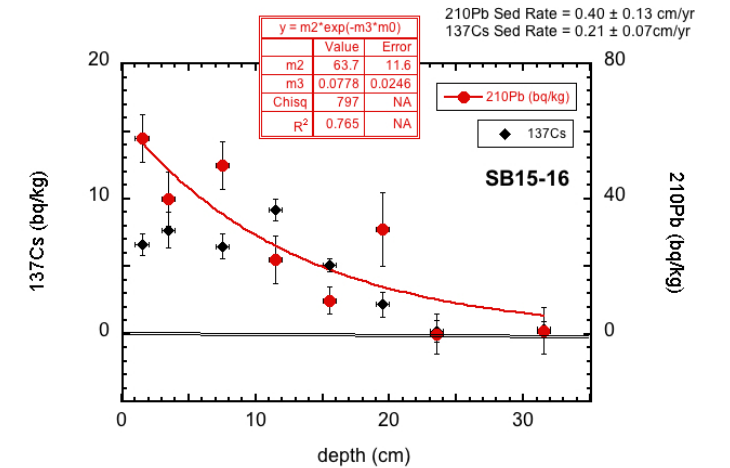

e) SB15-20

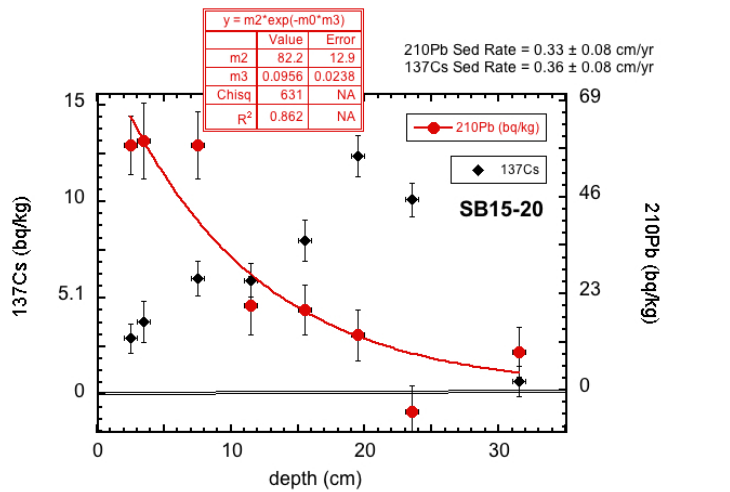

f) SB15-21

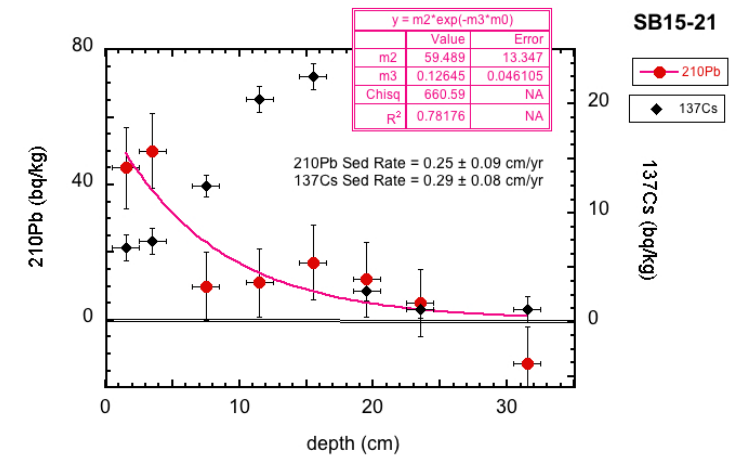

Supplement: Supplementary file 2 — Appendix Figure2 [file 267_2023_1897_MOESM2_ESM.pdf]
