# Supplementary material for: Increasing Salt Marsh Elevation Using Sediment Augmentation: Critical Insights from Surface Sediments and Sediment Cores
Source: Environ Manage. 2023 Nov 1;73(3):614–33. doi: 10.1007/s00267-023-01897-8 (PMC10884093; doi:10.1007/s00267-023-01897-8)
Supplement: Supplementary file 3 — Appendix_Table1 [file 267_2023_1897_MOESM3_ESM.docx]

**Table 1**. **Radiocarbon table**

| **Core** | **Depth (cm)** | **14C Age (BP) Uncalibrated** | **Error (BP)** | **Age (YBP)** | **Error (YBP)** |
| --- | --- | --- | --- | --- | --- |
| SB15-06 | 99 | 340 | 15 | 380 | 78 |
| SB15-09 | 110 | 995 | 20 | 931 | 27 |
| SB15-11 | 110 | 1060 | 15 | 956 | 25 |
| SB15-11* | 174 | 870 | 40 | 781 | 106 |
| SB15-16 | 91 | 925 | 15 | 875.5 | 36 |
| SB15-20 | 101 | 865 | 20 | 776 | 83 |
| SB15-20 | 166 | 1620 | 60 | 1502 | 126 |
| SB15-20* | 206 | 640 | 15 | 589 | 50 |
|  |  |  |  |  |  |
