# Supplementary material for: Increasing Salt Marsh Elevation Using Sediment Augmentation: Critical Insights from Surface Sediments and Sediment Cores
Source: Environ Manage. 2023 Nov 1;73(3):614–33. doi: 10.1007/s00267-023-01897-8 (PMC10884093; doi:10.1007/s00267-023-01897-8)
Supplement: Supplementary file 4 — Appendix [file 267_2023_1897_MOESM4_ESM.docx]

**Appendix**

**Appendix Figure Captions**

*Figure 1 Black and white*

**Figure 1** Bacon Age-Depth Models for a) SB15-06, b) SB15-09, c) SB15-11, d) SB15-16, e) SB15-20, and f) SB15-21.

**Figure 2** Lead and cesium curves for sediment cores a) SB15-06, b) SB15-09, c) SB15-11, d) SB15-16, e) SB15-20, and f) SB15-21.

**Table 1** Radiocarbon results, reported as uncalibrated ^14^C age before present (BP) from the University of California, Irvine Keck-Carbon Cycle AMS facility, as well as calibrated years before present (YBP).
